# Meta-Analysis of *Caenorhabditis elegans* Transcriptomics Implicates Hedgehog-Like Signaling in Host-Microbe Interactions

**DOI:** 10.3389/fmicb.2022.853629

**Published:** 2022-05-10

**Authors:** Alejandra Zárate-Potes, Irtiqa Ali, Margarida Ribeiro Camacho, Hayley Brownless, Alexandre Benedetto

**Affiliations:** Division of Biomedical and Life Sciences, Lancaster University, Lancaster, United Kingdom

**Keywords:** *C. elegans*, Hedgehog, host-microbe interactions, transcriptomics, RNAi, LFASS, infection, stress

## Abstract

Controlling nematode-caused diseases that affect cattle and crops world-wide remains a critical economic issue, owing to the lack of effective sustainable interventions. The interdependence of roundworms and their environmental microbes, including their microbiota, offers an opportunity for developing more targeted anthelminthic strategies. However, paucity of information and a currently narrow understanding of nematode-microbe interactions limited to specific infection contexts has precluded us from exploiting it. With the advent of omics approaches to map host-microbe genetic interactions, particularly in the model roundworm *Caenorhabditis elegans*, large datasets are now available across multiple models, that enable identification of nematode-microbe-specific pathways. In this work we collected 20 transcriptomic datasets documenting gene expression changes of *C. elegans* exposed to 20 different commensal and pathogenic microbes, performing gene enrichment analyses followed by functional testing using RNA interference directed toward genes of interest, before contrasting results from transcriptomic meta-analyses and phenomics. Differential expression analyses revealed a broad enrichment in signaling, innate immune response and (lipid) metabolism genes. Amongst signaling gene families, the nematode-divergent and expanded Hedgehog-like signaling (HHLS) pathway featured prominently. Indeed, 24/60 *C. elegans* Hedgehog-like proteins (HRPs) and 15/27 Patched-related receptors (PTRs) were differentially expressed in at least four microbial contexts, while up to 32/60 HRPs could be differentially expressed in a single context. interestingly, differentially expressed genes followed a microbe-specific pattern, suggestive of an adaptive microbe-specific response. To investigate this further, we knocked-down 96 individual HHLS genes by RNAi, using high-throughput assays to assess their impact on three worm-gut infection models (*Pseudomonas aeruginosa*, *Staphylococcus aureus*, and *Enterococcus faecalis*) and two worm-commensal paradigms (*Comamonas* sp., and *Bacillus subtilis*). We notably identified new putative infection response genes whose upregulation was required for normal pathogen resistance (i.e., *grl-21* and *ptr-18* protective against *E. faecalis*), as well as commensal-specific host-gene expression changes that are required for normal host stress handling. Importantly, interactions appeared more microbe-specific than shared. Our results thus implicate the Hedgehog-like signaling pathway in the modulation and possibly fine-tuning of nematode-microbe interactions and support the idea that interventions targeting this pathway may provide a new avenue for anthelmintic development.

## Graphical Abstract

Graphical abstract of the approach employed in this study. Combined analyses of C. elegans-microbe transcriptomics (1) and gene tissue expression maps (2), with high-throughput stress and infection assays following host-gene inactivation (3), can guide predictions and accelerate identification of genetic interactions involved in nematode-microbe interactions.

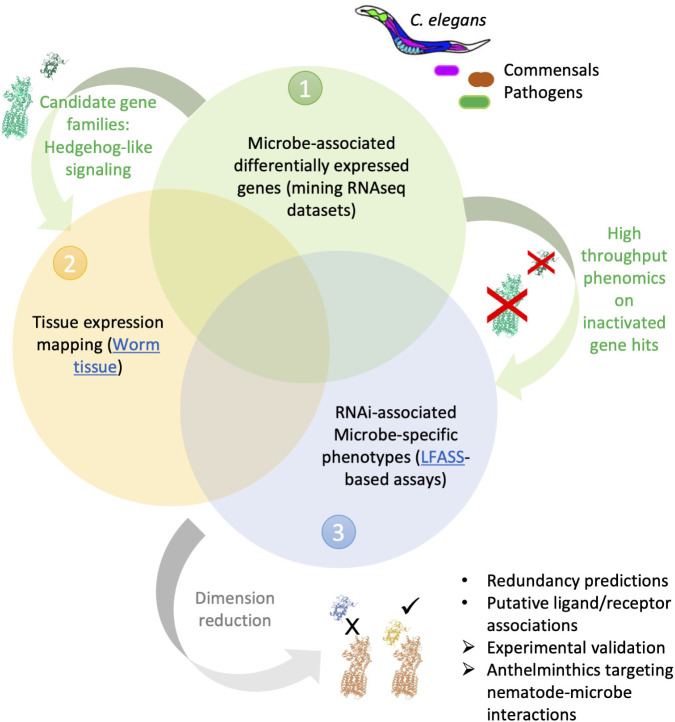

## Introduction

Parasitic nematodes represent a significant economic burden globally, responsible for neglected tropical diseases, cattle and pet diseases, and crop yield losses (Mitiku, 2018; Zajac and Garza, 2020). This has motivated the continuous development of anthelminthics and nematicides that target such parasites directly, yet many of them either lack specificity or potency, resulting in rapid evolution of resistance, or having sustained negative impacts on the environment (Doyle and Cotton, 2019; Wit et al., 2021). Nematodes have evolved alongside microbes, with which they entertain neutral, beneficial, and detrimental dynamic relationships that strongly impact their health. This realization opens the possibility of inhibiting or interrupting the nematode life cycle either by interfering chemically, or by using natural or bio-engineered microbiological agents that disrupt or hijack specific natural worm-microbe interactions.

As empirical approaches in *C. elegans* involving small-molecule screening have proven successful (Weicksel et al., 2016; Ikeda et al., 2020; Tjahjono et al., 2021), in an era where the number of omics datasets available is rapidly expanding, mining such resources for new nematode-specific druggable pathways looks increasingly promising (Coghlan et al., 2019). Currently, omics data available on nematode-microbe interactions largely come from studies performed on the non-parasitic soil roundworm *C. elegans*. This trend is expected to continue following the characterization and sequencing of the *C. elegans* gut microbiome and the ability to easily grow > 95% of *C. elegans* gut microbes in standard laboratory conditions (Berg et al., 2016; Dirksen et al., 2016; Samuel et al., 2016; Johnke et al., 2020). The nematode-specific evolution and expansion of specific gene families (Cox et al., 1984; Burglin and Kuwabara, 2006; O’Halloran et al., 2006; Burglin, 2008; de Abreu et al., 2014; Pees et al., 2016; Coghlan et al., 2019), the conservation of larval stage physiologies across roundworms, and previous research (Jasmer et al., 2020) indicate that meta-analyses of *C. elegans*-microbe datasets can yield critical information in our fight against parasitic roundworms.

A pathway of particular interest is the nematode divergent and expanded Hedgehog-like signaling (HHLS) pathway (Supplementary Figure 1) that produces 61 Hedgehog-related peptides (HRPs), 27 Patched-related receptors (PTRs) and two Dispatched orthologs in *C. elegans* (Burglin and Kuwabara, 2006; Burglin, 2008), and has also undergone expansion in additional free-living and parasitic nematode species (Supplementary Figure 1). Although the worm genome expresses a single Gli homolog (TRA-1) primarily involved in sex-determination (Hodgkin and Brenner, 1977; Hodgkin, 1987; Ellis, 2020), the nematode HHLS pathway lacks the canonical Hedgehog intracellular transduction pathway, and the evolutionary function of the PTR and HRP family expansions remains unclear (Baker et al., 2021). HHLS genes have been involved in *C. elegans* epithelial function and development (Hao et al., 2006a; Liegeois et al., 2006; Soloviev et al., 2011), cuticular structure and patterning (Zugasti et al., 2005; Hao et al., 2006b,c; Liegeois et al., 2006; Kouns et al., 2011; Chiyoda et al., 2021; Cohen et al., 2021), sensory organ formation (Michaux et al., 2000; Liegeois et al., 2007; Oikonomou et al., 2011; Wang et al., 2017; van der Burght et al., 2020), neurogenesis (Kume et al., 2019) and axonal guidance (Riveiro et al., 2017), immunity (Roberts et al., 2010; Lightfoot et al., 2016), lipid homeostasis (Lin and Wang, 2017; Del Castillo et al., 2021), reproduction (Kuwabara et al., 2000; Templeman et al., 2020), and longevity (Ji et al., 2021). In particular, the HHLS pathway was found to mediate the impact of microbial metabolites on host lipid metabolism (Lin and Wang, 2017), supporting a direct role in host-nematode interactions that could in part explain the diversification of HRP ligands and PTR receptors.

Looking for evidence of a broader involvement of the HHLS pathway in nematode-bacterium interactions, we mined a compilation of *C. elegans* RNAseq datasets published, and recently generated in our lab. We performed a meta-analysis comparing the worm’s transcriptomic response to a wide range of microbes (pathogens, probiotics, commensals, prokaryotic and eukaryotic), reporting on immune and metabolic gene expression changes, before focusing on the HHLS pathway. To determine whether Hedgehog-like signaling plays a role in *C. elegans*-gut pathogen interactions, we subjected worms with RNAi-impaired HHLS to Gram-positive (G +) (*E. faecalis*, *S. aureus*) and Gram-negative (G–) (*P. aeruginosa*) pathogens. We next tested the ability of worms with impaired HHLS to establish homeostatic relationships with gut commensals, by exposing them to commensal G + vs. G- bacteria before challenging them with heat or oxidative stress. Our results reveal a complex picture that supports a role for multiple HHLS genes in host-microbe interactions.

## Materials and Methods

### *Caenorhabditis elegans* and Bacterial Culture and Strains

Supplementary Table 1 contains a list of all *C. elegans* and bacterial strains used in this study. We used the *C. elegans* NL2099 *rrf-3*(*pk1426*) II in our assays for its increased sensitivity to RNAi (Simmer et al., 2002), but otherwise wild type behavior and life traits. Worms were maintained on nematode growth medium (NGM) agar plates at 15°C and fed with bacterial lawns of *E*. *coli* OP50 as described (Stiernagle, 2006). For experiments, *C. elegans* were transferred to fresh 15 cm diameter NGM plates inoculated with 3 mL *E*. *coli* OP50 and 1 mL concentrated *E*. *coli* OP50 and grown at 20°C until a sufficiently large population was obtained. Gravid *C. elegans* populations were washed from plates with M9 buffer (KH_2_PO_4_ (22 mM), Na_2_HPO_4_ x 2 H_2_O (33.7 mM), NaCl (85.6 mM), supplemented with 1 mL/L MgSO_4_ (1 M) after autoclaving) and synchronized by bleaching: 8 mL washed worms/eggs in M9 were mixed with 2 mL of a 1:1 solution of NaOH (4 M) and NaClO (12%) for 7 min followed by vortexing and inverting. Bleaching was stopped by centrifuging for 2 min at 1,550 rpm, supernatant was discarded, and pellet was washed two times with 10 mL M9 buffer and 2 times with 10 mL autoclaved MiliQ water. Only eggs survived the treatment. An extra synchronization step included incubation of bleached eggs in uninoculated 15 cm NGM plates at 20°C for 24 h until all eggs had hatched. Synchronized L1 larval stage *C. elegans* were subsequently used for experiments.

*E. coli* OP50 was grown at 37°C with shaking overnight in OP50 liquid medium (5 g Tryptone and 2.5 g of Yeast Extract per Liter of MiliQ water). Concentrated *E. coli* OP50 cultures for worm feeding were prepared by inoculating 1 L of Luria-Bertani (LB) broth (Invitrogen) with 10 mL of overnight *E. coli* OP50 culture and grown for 4 h (or until saturated) at 37°C with shaking. Saturated cultures were centrifuged at 3,500 rpm for 15 min at room temperature, supernatants were discarded and pellets of 6 liters of cultures were collected in 9 mL OP50 medium. The resulting concentrated *E. coli* OP50 culture was used directly to inoculate NGM plates. *Enterococcus faecalis* OG1RF was streaked from frozen stocks on Brain Heart Infusion (BHI) (Sigma-Aldrich) agar plates and grown at 37°C overnight. Liquid cultures were grown on BHI broth for 3 h at 37°C and used to inoculate Nematode Growth BHI plates (NGBHI) (BHI agar plates supplemented with 2 g of agar per liter to match the agar concentration of NGM). Matching *E. coli* OP50 controls were grown in the same way. *Pseudomonas aeruginosa* PA14, *Staphylococcus aureus* 6538 and *Bacillus subtilis subsp. subtilis* 168 were maintained on LB agar and broth and cultured at 37°C. *Comamonas* sp. B-9 MYb021 was maintained on LB agar and broth and cultured at 25°C.

### Mining Published *Caenorhabditis elegans* Transcriptomic Datasets

To study the transcriptional response of *C. elegans* to microbial exposure we collected published RNAseq datasets that report differential gene expression in *C. elegans* when exposed to different microorganisms as a Fold Change (FC) (which were converted to Log_2_FC) or Log_2_FC and report an adjusted *p*-value. To find these publications we used the online search engine Research Rabbit,^1^ which was developed to help find across the web additional publications like the publications on a user-made preselected list. The detailed list of datasets collected is summarized in Supplementary Table 2 and the collected data of differential gene expression and adjusted *p*-values can be found in Supplementary Table 4 reported as Log2FC and adjusted *p*-values. Further analyses and graphing were performed with RStudio^2^ using the *C. elegans* gene functional annotation available from WormCAT (Holdorf et al., 2020). The code to produce bubble plots of lists of genes of interest with our collection of datasets is available on GitHub. As published datasets were not independently re-analyzed from raw data, or re-scaled, datasets from different studies cannot be compared quantitatively, only qualitatively.

### Sequence Search, Phylogenetic Trees, and Statistical Analyses in R

HHLS paralogs in nematodes were identified in Wormbase Parasite by performing a cDNA TBLASTN search for distant homologs to *C. elegans* WRT-1, WRT-8, GRD-1, GRL-1, GRL-7 (ligands) and PTR-1, PTR-18, PTC-1, CHE-14 (receptors) followed by species per species paralog search (numbers of paralogs may be underestimated). Percentage identities of sequences were obtained using BLASTP or TBLASTN of *C. elegans* proteins against target species databases. For phylogenetic analyses amino acid sequences of *C. elegans* Hedgehog-like (HHL) pathway genes and modulators and human sonic hedgehog (SHH), patched (PTC1) and dispatched (DISP1) were downloaded using QIAGEN CLC Main Workbench 20.0.^3^ Sequences were aligned using the default parameters of ClustalW. Phylogenetic trees were constructed using the Neighbor-Joining method with 1,000 Bootstrap repetitions assuming the Jukes-Cantor amino acid evolution model.

### RNAi Screening of Hedgehog-Like Signaling Pathway Genes

Worms were grown at 20°C on 15 cm NGM plates inoculated with *E. coli* OP50 and concentrated *E. coli* OP50 until the population size was large enough for the desired experiments. The parental worm population was bleached as described above. To test the functional involvement of HHL pathway genes in resistance to stress and infection we performed target gene knockdown by feeding *C. elegans* NL2009 L1 larvae with the RNAi clones form the Source Bioscience Ahringer library (Kamath et al., 2003) listed in Supplementary Table 1. RNAi was performed in a 96-well plate array of NGM supplemented with 50 μg/mL Carbenicillin and 2 mM Isopropyl-β-D-thiogalactoside (IPTG) (Sigma-Aldrich) inoculated with concentrated RNAi bacteria. RNAi bacteria were cultured overnight at 37°C with shaking (200 rpm) in 96-deep well plates with 1.5 mL Terrific Broth [Tryptone (12 g), yeast extract (24 g), glycerol (100% 4 mL) in 900 mL MiliQ H_2_O, autoclaved and then mixed with 17 mL KH_2_PO_4_ (1 M) and 72 mL K_2_HPO_4_ (1 M)], supplemented with 100μg/mL Ampicillin and 0.2 mM IPTG. Over 100 *C. elegans* larvae per well were cultured on RNAi bacteria at 20°C until reaching L4 larval stage. Subsequently they were transferred to 25°C for 24 h until they reached young adult stage and were able to take up live bacteria in their guts.

For the stress assays RNAi-fed worms were transferred to further NGM 96-well plates inoculated with 15 μL of a concentrated overnight liquid culture of a G + (*Bacillus subtilis* subsp. Subtilis 168) and a G- (*Comamonas* sp. B-9 MYb021) *C. elegans* commensal microbe, for 24 h at 25°C. Stress and infection assays were performed by pipetting 30 μL of M9 buffer to each well of the 96-well plates and collecting 20 μL of worms per well, which were pipetted to individual wells of a 384 black well plate with clear bottom. For stress assays each sample was done in duplicate with three independent runs and for infection assays each sample was done in tetraplicate with three independent runs and no exposure to commensal bacteria. For the heat sock assays M9 was added to a final volume of 67 μL per well and worms were treated at 42°C for 12 h. For the oxidative stress assays 40 μL of M9 and 7 μL of tert-Butyl hydroperoxide (TBHP) 70% in H_2_O (Sigma-Aldrich) were added to a final volume of 67 μL and final concentration of 7.3% TBHP per well and worms were treated at 25°C for 12 h. For the infection assays RNAi-fed worms were kept at 25°C for a total of 36 h until they reached day one of adulthood and then pipetted to 384-well plates. For infections, *E. faecalis* OG1RF was grown for 3 h in 5 mL BHI broth, *P. aeruginosa* PA14 and *S. aureus* 6538 were grown overnight in 5 mL LB broth at 37°C with shaking. To the 384-well plate 20 μL of M9, 20 μL of OP50 medium and 7 μL of liquid pathogen culture were added to a final volume of 67 μL per well and worms were treated at 25°C for 60 h.

### Label-Free Automated Survival Scoring Analyses

Median time of death was determined by Label-Free Automated Survival Scoring (LFASS) analysis (Benedetto et al., 2019) of death fluorescence recordings (Exc./Em.: 365 nm/430 nm) in Spark, Infinite Pro M200, or MNano + plate-readers (Tecan). Data were processed in MATLAB 2020a (MathWorks), exported to Excel 365 (Microsoft Office), and processed for graph plotting and statistical analyses in Prism 9.3 (GraphPad). Two-way ANOVA were performed with *post hoc* Tukey correction for multiple comparisons.

## Results

### Published *Caenorhabditis elegans* Transcriptomics Datasets Allow for the Study of Gene Expression Changes After Exposure to Diverse Microbial Species

**FIGURE 1:**
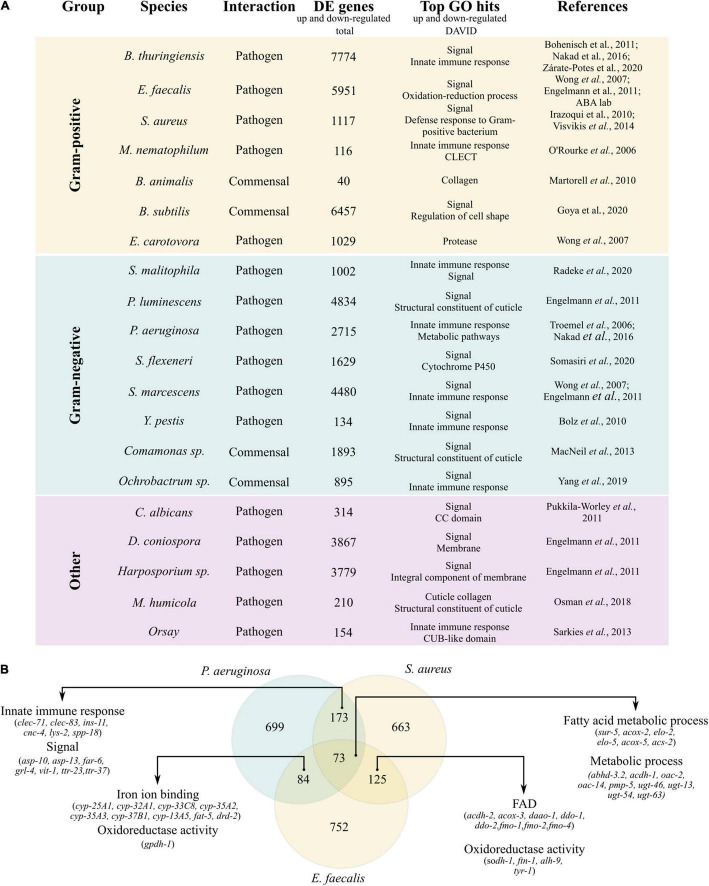
Gene expression changes of *C. elegans* exposed to diverse microbes. **(A)** Shows a list of 20 different microbes for which gene expression data of exposed *C. elegans* is available. The table includes: The nature of the interaction with *C. elegans*, either pathogenic or commensal; the total number of unique significantly differentially expressed (DE) genes combining all different time points and datasets; the top two hits of gene set enrichment analysis performed with the online tool DAVID (https://david.ncifcrf.gov/) using the unique set of DE genes per microbe (both up- and down-regulated together) (the complete results of the enrichment analysis can be found in Supplementary Table 3); and the references from which the datasets were obtained. In **(B)** a Venn diagram showing the overlaps between the list of unique DE genes of *C. elegans* exposed to *P. aeruginosa*, *E. faecalis*, and *S. aureus*. The top two hits of the enrichment analysis using DAVID and representative genes for each enrichment term are shown. A full gene ID list and the full results of the enrichment analysis are shown in Supplementary Table 3.

Our literature search identified 20 published accessible transcriptomic datasets on wild type *C. elegans* exposed to microbes, to which we added an unpublished RNAseq dataset of *C. elegans* exposed to *E. faecalis* OG1RF generated in our lab (publication in preparation). This collection includes differential gene expression data for *C. elegans* exposed to 20 different microorganisms including pathogenic and non-pathogenic strains of Gram-positive (G +) bacteria, Gram-negative (G-) bacteria, yeast, fungi, microsporidia, an oomycete, and Orsay virus (Figure 1A and Supplementary Table 2). For each different microbe we extracted a list of unique significantly differentially expressed (DE) genes (up- and down-regulated) combining all available time points and datasets, for which we performed enrichment analysis using the online tool DAVID (Figure 1A and Supplementary Table 3).

For both pathogenic and commensal microbe exposures, DE genes were enriched in genes whose function contains the keyword “signal” (Figure 1A). Genes annotated with this keyword notably include those coding for the nematode divergent and expanded families of C-type-lectin-like domain-containing proteins (CLECs) and the Hedgehog-like signaling (HHLS) pathway. DE gene lists were also enriched in genes relating to defense against stress or pathogens, innate immune function, xenobiotic metabolism (Cytochrome p450), structural proteins (collagens), and enzymes (proteases, oxidation-reduction processes, metabolism) (Figure 1A and Supplementary Table 3). Overlaps between all gene lists are shown in Supplementary Table 5. Ranking of individual genes based on the number of datasets in which there were found differentially expressed (considering each condition as a different dataset, Supplementary Figure 2 and Supplementary Table 5) confirmed the importance of lipid metabolism in host-microbe interactions. The top two genes, found differentially expressed in 35 out of 56 datasets, were the Acyl CoA Dehydrogenase *acdh-1*, involved in beta-oxidation (lipid metabolism), and the folate transporter family member *folt-2*, involved in transmembrane transport (Supplementary Figure 2 and Supplementary Table 5). While 19 out of 75 DE genes found in 26/56 datasets were expectedly associated with “stress response: Pathogens,” genes associated with lipid metabolism were the next most highly represented category (11/76, Supplementary Table 5).

For easier follow-up of the genes highlighted by our enrichment analyses, we narrowed further analyses to: (1) Candidates gene families with the potential of modulating specific interactions with microbes, (2) experimental models that are amenable to high-throughput testing. For this, we focused on genes that are DE in the context of three well-documented gut bacterial infection models (G + and G- bacteria) that are readily available, where a breadth of resources already exist, and for which high-throughput infection assays with convenient readouts have been established: *Pseudomonas aeruginosa* PA14 (Mahajan-Miklos et al., 1999), *Staphylococcus aureus* 6538 (Sifri et al., 2003), and *Enterococcus faecalis* OG1RF (Garsin et al., 2001). DE gene lists for these three microbes, *E. faecalis* OG1RF (5,951 genes), *P. aeruginosa* PA14 (2,715 genes), and *S. aureus* 6538 (1,117 genes combined from datasets from *S. aureus* strains RN6390 and CECT8145) highlighted a small subset of 73 shared genes predominantly belonging to the functional grouping “fatty acid metabolism” (Figure 1B and Supplementary Tables 3, 5). Collectively the results of these enrichment analyses and summary counts of gene expression data show that new microbial exposure in *C. elegans* broadly leads to modulation of signal molecules, immune and metabolic (lipids) genes, in line with previously published studies.

### Metabolic and Immune Pathways Are Modulated in a Microbe-Specific Manner

**FIGURE 2:**
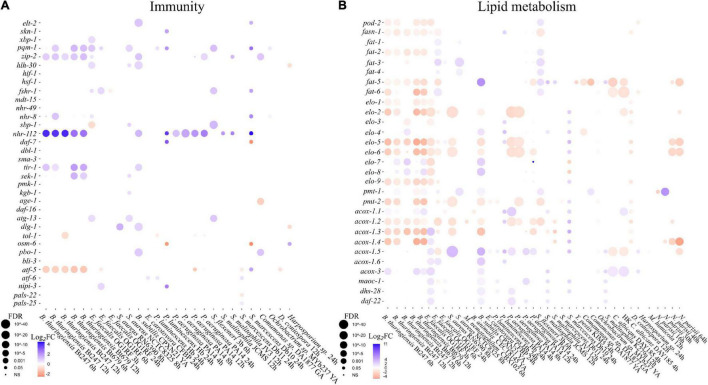
*C. elegans* immune regulation and lipid metabolism genes change their expression in a microbe-specific manner. Gene expression plots (bubble plots) that represent changes in gene expression as log_2_Fold Change (Log_2_FC) in a color heatmap and the adjusted *p*-value (FDR) as the diameter of the points. Published transcriptomic datasets were not re-analyzed from raw data, or re-scaled, therefore datasets from different studies cannot be compared quantitatively, only qualitatively. Detailed information about the datasets shown can be found in Supplementary Table 2. **(A)** Shows gene expression changes in a list of selected immunity genes (Pukkila-Worley and Ausubel, 2012; Martineau et al., 2021). **(B)** Shows gene expression changes in a list of selected lipid metabolism genes (Watts and Ristow, 2017).

To highlight specific molecular pathways, we next characterized how the expression of genes changes in those categories during microbial exposure across all 56 datasets. From the published literature, we extracted genes of interest involved in *C. elegans* immune signaling and regulation (Pukkila-Worley and Ausubel, 2012; Martineau et al., 2021) and lipid metabolism (Watts and Ristow, 2017). We then used these lists to query the collected transcriptomic datasets (Supplementary Table 4) and produced bubble plots representing differential gene expression (Log_2_FC) and adjusted *p*-values (FDR) for the relevant datasets (Figure 2). Immune signaling and modulation genes were generally upregulated across a wide variety of microbes with some exceptions, including the putative cAMP-dependent transcription factor *atf-5* and the predicted signal receptor *tol-1.* Interestingly, the robust upregulation of the nuclear hormone receptor *nhr-112* across a variety of datasets suggests a significant role for this transcription factor in interactions with G + and G-negative pathogens and commensals alike, which had not been reported. Conversely, several known defense and stress response modulators, such as the FOXO transcription factor *daf-16*, failed to exhibit significant changes in gene expression in either of the datasets. Despite the established role of DAF-16 in immune modulation, this was not unexpected as modulation of DAF-16 activity mostly occurs post-translationally, via regulated shuttling between the cytosol and the nucleus (Lee et al., 2001; Figure 2A).

By contrast with known conserved immune regulators, lipid metabolism genes were more extensively differentially expressed following microbial exposure (Figure 2B). Genes involved in *de novo* fatty acid metabolism (*pod-2, fasn-1, fat-1-6, elo-1-7, pmt-1-2*) or lipid catabolism (*acox, maoc-1, dhs-28 and daf-22*) (Watts and Ristow, 2017) were broadly down-regulated following exposure to the G + pathogens *B. thuringiensis, E. faecalis* and *S. aureus*, and the G- pathogen *P. aeruginosa* (Figure 2B).

Interactions between fatty acid metabolism and innate immunity (Anderson and Pukkila-Worley, 2020) or between fatty acid metabolism and Hedgehog-like signaling (Lin and Wang, 2017; Del Castillo et al., 2021) have been frequently reported in animals, including in *C. elegans*. Increased *C. elegans* fatty acid metabolism is also strongly associated with reproduction (Ezcurra et al., 2018), while a trade-off between reproduction and the mounting of effective innate immune responses has been previously established (TeKippe and Aballay, 2010). That DE fatty acid metabolism genes are mostly down-regulated upon pathogen exposure (Figure 2B), while DE innate immunity genes are up-regulated (Figure 2A) is consistent with that. However, the core immunity and lipid metabolism genes identified above represent a small fraction of the DE genes for any given microbe (Figure 1B), highlighting the fact that the transcriptional modulation of most DE genes is microbe specific. We wondered whether such specificity might be underpinned by the finely tuned expression of specific classes of signaling peptides and cognate receptors families such as the expanded HHLS network.

### The *Caenorhabditis elegans* Hedgehog-Like Signaling Pathway Is Modulated by Microbes and Impacts Resistance to Pathogens in a Microbe-Specific Manner

The top hits of our gene ontology analysis related to “Signal,” which included a high proportion of HRPs. The remarkable non-redundant expansion of the HHLS pathway ligands and Patched-related receptor (PTRs) families in nematodes (Zugasti et al., 2005; Burglin, 2008; Baker et al., 2021; Supplementary Figure 1) and the conserved and intricate roles of Hedgehog pathway genes in intercellular signaling throughout metazoa (Ingham et al., 2011) make the non-canonical (Jenkins, 2009) nematode HHLS pathway a prime candidate for enabling specificity in host-microbe interactions. We thus investigated to which extent HHLS genes and known regulators were differentially expressed in worms exposed to a diversity of microbes, and if downregulation of these genes could disrupt interactions between the worm host and cognate microbes.

**FIGURE 3:**
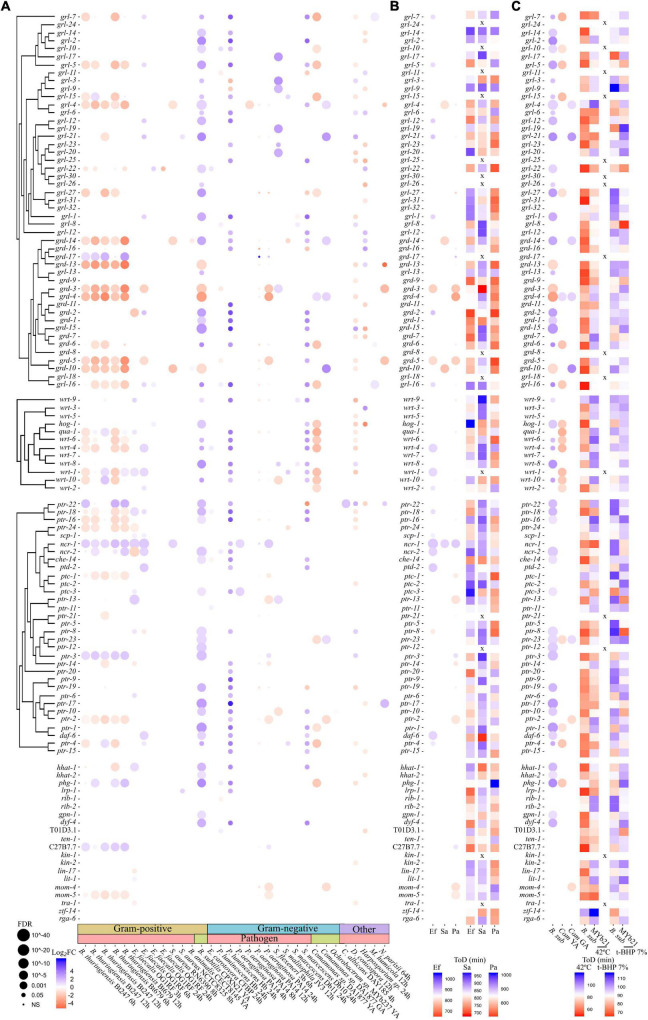
In *C. elegans* Hedgehog-like (HHL) genes and genetic interactors play microbe-specific roles in the defense against infection and microbe-mediated protection from stress. **(A)** Bubble plots representing gene expression changes in Hedgehog-like (HHL), patched-related receptor (PTR) and hedgehog genetic interactors after exposure to different microbes. Bubble plots represent changes in gene expression as log2Fold Change (Log_2_FC) in a color heatmap and the adjusted *p*-value (FDR) as the diameter of the points. Published transcriptomic datasets were not re-analyzed from raw data, or re-scaled, therefore datasets from different studies cannot be compared quantitatively, only qualitatively. **(B)** Heatmaps showing results of mean time of death (estimated by LFASS) after infection with *P. aeruginosa* (Pa), *E. faecalis* (Ef), and *S. aureus* (Sa). Bubble plots showing gene expression changes after exposure to the same microbes is repeated for comparison. **(C)** Heatmaps showing results of mean time of death (estimated by LFASS) after exposure to deadly oxidative stress 7% t-BHP and heat stress (42°C). Before the stress assays, RNAi-treated worms were exposed for 24 h at 25°C to commensal G- (*Comamonas* sp.) and G + (*B. subtilis*) stress-protective bacterial isolates. Bubble plots showing gene expression changes after exposure to *Comamonas* sp. and *B. subtilis* is repeated for comparison. x represents genes not included in the RNAi screen. YA, Young Adult, GA, Gravid Adult.

Surveying the datasets collected (Figure 3A), out of 61 *C. elegans* HRPs from the GRL/GRD/WRT/QUA/HOG families 31 were found differentially expressed (27 up-regulated) on *B. subtilis*, 29 (27 up-regulated) on *P. luminescens*, 25 (22 up-regulated) on *Serratia marcescens*, 21 (20 down-regulated) on *B. thuringiensis*, 18 (14 down-regulated) on *Comamonas* sp., 13 (11 up-regulated) on *E. faecalis*, 9 (8 up-regulated) on *S. flexneri*, 5 (3 up-regulated) on *S. malitophila*, 4 (3 down-regulated) on *P. aeruginosa*, and 4 (3 down-regulated) on *Staphylococcus aureus*. PTR expression followed the same trend as HRP expression (where HRP genes were mostly down-regulated, PTR genes were mainly down-regulated as well), suggesting microbe-specific purposeful activation or inhibition of HHLS altogether. G + and G- commensals and pathogen types elicited differential expression of HHLS pathway genes, with no obvious type-specific pattern beyond the fact that G + bacteria seemed to elicit more obvious shifts in HHLS gene expression in this dataset. Most changes appear to be species or condition specific. HHLS genes were also differentially expressed following exposure to pathogenic fungi such as *D. coniospora* (26 DE, 19 down-regulated) and *Harposporrium* sp. (19 DE, 11 down-regulated) and microsporidium *Nematocida parisii* (6 DE, 3 down-regulated), but not after viral infection by the Orsay virus (no hits—not represented in Figure 3A). Although most HHLS pathway genes did not follow that pattern, some sequence-related genes were co-regulated upon exposure to multiple microbes (Figure 3A, *grd-3/grd-4*, *qua-1/wrt-6/wrt-4*, *wrt-1/wrt-10*, *ptr-16*, and *ptr-18*), in line with their tissue expression patterns (Kaletsky et al., 2018; Supplementary Figure 3), which might indicate functional redundancy.

**FIGURE 4:**
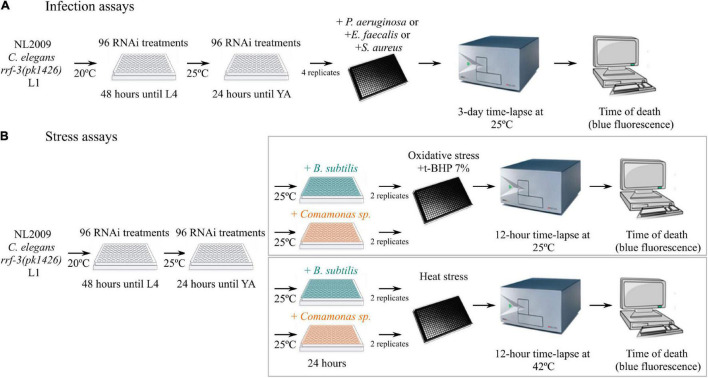
Schematic representation of approach to screen Hedgehog-like (HHL) genes and genetic interactors by RNAi inactivation. For all assays the RNAi-sensitive *C. elegans* strain NL2009 was used. Time of death was detected by blue death fluorescence as established in the Label-Free Automated Survival Scoring (LFASS) method (Benedetto et al., 2019). **(A)** Shows details of the approach for infection with *P. aeruginosa*, *E. faecalis*, and *S. aureus* and **(B)** shows details of the approach for the oxidative stress assay with 7% t-BHP and the heat stress assay at 42°C. Before the stress assays, RNAi-treated worms were exposed for 24 h at 25°C to commensal G- (*Comamonas* sp.) and G + (*B. subtilis*) stress-protective bacterial isolates. YA, Young Adult.

As changes in expression levels may not translate into functional changes, we next targeted HHLS pathway genes one by one from the late L1 larval stage using RNAi-expressing clones from the Ahringer library (Kamath et al., 2003) in the RNAi-sensitized strain *rrf-3(pk1426)*(Simmer et al., 2002), and assessed the impact of this treatment on subsequent resistance to infections by three model gut pathogens: *P. aeruginosa* PA14 (Mahajan-Miklos et al., 1999), *S. aureus* 6538 (Sifri et al., 2003), and *E. faecalis* OG1RF (Garsin et al., 2001; Figures 3B, 4A and Supplementary Table 6). Amongst the 73 DE genes common to all three pathogen infections, only *ncr-1* [homolog of the vertebrate gene Niemann-Pick disease, type C1 (NPC1)], a sterol-sensing domain-containing protein involved in cholesterol trafficking (Patel et al., 2008) can be associated with HHLS (Figure 3B), as most gene expression changes in the HHLS pathway seem pathogen-specific. This may not be surprising when considering that together with *ptr-5*, *ncr-1* is the most ubiquitously expressed SSD-protein in *C. elegans* tissues (Supplementary Figure 3). Our infection assays also indicated that RNAi against individual HHLS genes and genetic interactors differentially impacts *C. elegans* resistance to pathogens (Supplementary Figure 4) but revealed broader trends. Hence, inhibition of HHLS genes was generally found associated with reduced resistance to the G- pathogen *P. aeruginosa*, while the picture is more contrasted for the G + pathogens *E. faecalis* and *S. aureus* (Figure 3B).

Compared to *S. aureus* (4) and *P. aeruginosa* (12), worms exposed to *E. faecalis* exhibited more (20/87) differentially expressed HHLS ligands and receptors, most of them up-regulated (17/20). Interestingly, gene per gene comparison of expression data and RNAi-associated pathogen resistance reveals that several of these up-regulated genes (9/16 tested) are necessary for wild-type level resistance to *E. faecalis* infection, as RNAi against these led to increased worm susceptibility (Figure 3B). Among them are HRPs: *grl-21* (previously identified as a mediator of environment-host-microbe crosstalk; Lin and Wang, 2017), *grl-5*, *grl-7*, *grd-12*, *grd-2*, and *wrt-4*, and PTRs: *ptr-18* (required for HRP clearance; Chiyoda et al., 2021) and *daf-6* (necessary for sensory organ morphogenesis together with *dyf-4* (Hong et al., 2021), and thus possibly for pathogen sensing), as well as another SSD-protein expressing gene: *scp-1*. Similarly, RNAi inhibition of the *P. aeruginosa*-up-regulated *ncr-1* and C27B7.7 (DSCAM) led to increased host sensitivity to infection, implicating both genes in the worm immune response to *P. aeruginosa* gut infections, while RNAi inhibition of *ncr-1* (only gene found up-regulated in this context) did not sensitize worms to *S. aureus* infection. Conversely, the RNAi inhibition of several genes found downregulated upon exposure to pathogens led to increased resistance to infection. These genes include *ptr-2* and the MAPKKK *mom-4* and Frizzled ortholog *mom-5* in *P. aeruginosa* infection; *ptr-8* in *E. faecalis* infection and *grl-4* and *grd-14* in *S. aureus* infection (Figure 3B).

Across all three infection models, more RNAi tested had an adverse effect on host resistance to infection than there were up-regulated genes. The role of corresponding non-DE genes in pathogen resistance might be indirect, or regulation of their activity may primarily occur post-transcriptionally. This is expected as HRPs and PTRs activities in flies and mammals strongly rely on post-translational modifications and regulated intracellular trafficking (Carballo et al., 2018). Nevertheless, the widespread microbe-specific impact of HHLS gene inactivation on pathogen resistance suggests an active role for this pathway in response to pathogens, which may be pathogen-specific.

### Hedgehog-Like Signaling Is Modulated by Commensals and Impacts Host Stress Resistance in a Microbe-Dependent Manner

If the worm HHLS pathway is engaged in pathogen-specific responses, it may also be involved in the regulation of commensal-host interactions. We saw earlier (Figure 3A) that *C. elegans* exposure to commensal bacterium *B. subtilis* or to *P. luminescens* (which is a natural gut commensal of entomophagous nematodes; Ogier et al., 2020) leads to the up-regulation of many HHLS genes, while another commensal—*Comamonas* sp., leads to the down-regulation of most differentially expressed HHLS genes. We thus wondered whether these opposite effects on HRP and PTR gene expression levels are adaptive, enabling worms to tune Hedgehog-like signaling accordingly to the commensal they interact with. To test this idea, we grew worms from late L1 larval stage onto RNAi-producing dietary *E. coli* bacteria targeting HHLS genes, then transferred them as day 1 adults onto *B. subtilis* and *Comamonas* sp. (MYb21) isolates for 24 h, before challenging them with oxidative and thermal stresses (Figure 4B). Our expectations were that if a HHLS gene up-regulation was adaptative to a specific commensal, its down-regulation by RNAi would disrupt the relationship *C. elegans* normally establishes with that commensal, which would be revealed by increased frailty and sensitivity to oxidative or heat stress.

Analysis of stress assay data (Supplementary Figure 5) first revealed that oxidative stress (7% t-BHP) resistance is generally little affected by RNAi pre-treatment, while host heat-stress resistance is strongly impacted by both the RNAi applied and the nature of the microbial isolate considered. This may be related to the fact that heat-shock resistance appears to be a better proxy for adult worm health than oxidative stress resistance (Benedetto et al., 2019). Yet, RNAi toward individual HHLS genes differentially affected oxidative and heat stress resistance in a microbe-specific manner, with worms grown on different microbes exhibit strikingly different RNAi-associated stress resistance patterns. Worms exposed to *B. subtilis* displayed a wide-ranging up-regulation of HHLS genes, which was mirrored almost perfectly gene-by-gene by a negative impact of corresponding RNAi on heat resistance (Figure 3C). Indeed, RNAi down-regulation of 36 genes found up-regulated in worms grown on *B. subtilis* adversely affected the ability of worms to tolerate heat, while only 7 did not follow this rule (6 of which were found down-regulated in transcriptomics datasets and their down-regulation by RNAi did not translate into increased heat resistance). Conversely, for worms exposed to *Comamonas* sp. MYb21, which mostly experienced a down-regulation of HHLS genes, RNAi against these genes was as often beneficial as detrimental (9 and 8 genes, respectively). The impression emerging from these data is that while up-regulation of HHLS genes may be indicative of a “purposeful” adaptive response to a commensal, down-regulation of these genes may not be.

## Discussion

In this article we sought to combine (1) mining of publicly available transcriptomic datasets of *C. elegans* exposed to pathogenic and commensal microbes, with (2) new high-throughput phenotyping assays for probing host-microbe interactions, to identify nematode-specific genetic pathways that are potential targets for anthelminthic development (Graphical Abstract). We reasoned that the reliance of nematodes on entertaining adequate relationships with commensal and pathogenic microbes could offer an alternate, more specific pathway for disrupting nematode physiology. We began by collecting recently generated, available, transcriptomic datasets of *C. elegans* exposed to a diversity of microbes. We generated unified gene lists of differentially expressed (DE) genes for each microbe and performed enrichment analyses (Figure 1) that revealed a predominance of “signaling” genes belonging to several extended gene families, amongst which the elusive, non-canonical *C. elegans* Hedgehog-like signaling (HHLS) pathway. We contrasted HHLS gene expression changes upon exposure to microbes (Figures 1, 3A) with their tissue expression patterns (Supplementary Figure 3), and with the effects of individual RNAi targeting these genes on both: (1) Worm resistance to three gut bacterial pathogens (Figure 3B) and (2) the ability of worms to maintain a resilient relationship with two commensal gut bacteria. To probe the latter, we transferred worms grown on RNAi to commensal isolates before subjecting them to oxidative and thermal challenges (Figure 3C).

Our combined transcriptomic and survival assay data establish a role for HHLS genes in nematode-microbe interactions in the context of both pathogens and commensals. It identifies specific HRPs and PTRs that are differentially expressed in a microbe-specific manner and mediate effects on host health in the contexts of infections and commensalism. They also paint a complex picture that is difficult to decipher with the published data at hand, and current omics approaches. Firstly, most omics datasets are collected at a few discrete time intervals, which inevitably fails to capture the dynamics of host-microbe genetic interactions, where microbes and host sequentially respond to one another until either “loses” or a *status quo* is established. Depending on sampling timing, one dataset may reflect an unspecific, adaptive, or “microbe-coerced” host response, which can highlight genes expression changes that are advantageous to the host or to the microbe, confounding interpretations. However, as challenges for dual sequencing of host and microbial genes are progressively lifted (Westermann and Vogel, 2021), such datasets will help establish stronger associations between microbial activity and host response, helping identify the most relevant expression change. As exemplified in this paper, combination of omics establishing genetic links with high-throughput phenotyping assays to interrogate function is another increasingly popular approach that offers an affordable work-around (Partridge et al., 2018; Benedetto et al., 2019; Pryor et al., 2019). Secondly, HHLS is systemic. As such, functional changes associated with shifts in gene expression need to consider temporal and location information about the tissue in which these changes occur, to understand how information flows or is modulated, and what it means in a specific context. Indeed, HHLS is active throughout the development of the worm (Hao et al., 2006c; Kolotuev et al., 2009; Kouns et al., 2011; Oikonomou et al., 2011; Riveiro et al., 2017; Kume et al., 2019; Hong et al., 2021) and into aging (Templeman et al., 2020; Ji et al., 2021), it concerns a variety of HRPs and PTRs with specific tissue-expression patterns (Supplementary Figure 3) and non-redundant roles (Zugasti et al., 2005; Hao et al., 2006b; Baker et al., 2021). Tissue-specific omics datasets would thus greatly help disentangle the contributions of major tissues expressing HRPs and PTRs such as the nervous system, the hypodermis, the intestine, and the gonads, and of HHLS genes found co-expressed in several of them (Supplementary Figure 3).

**FIGURE 5:**
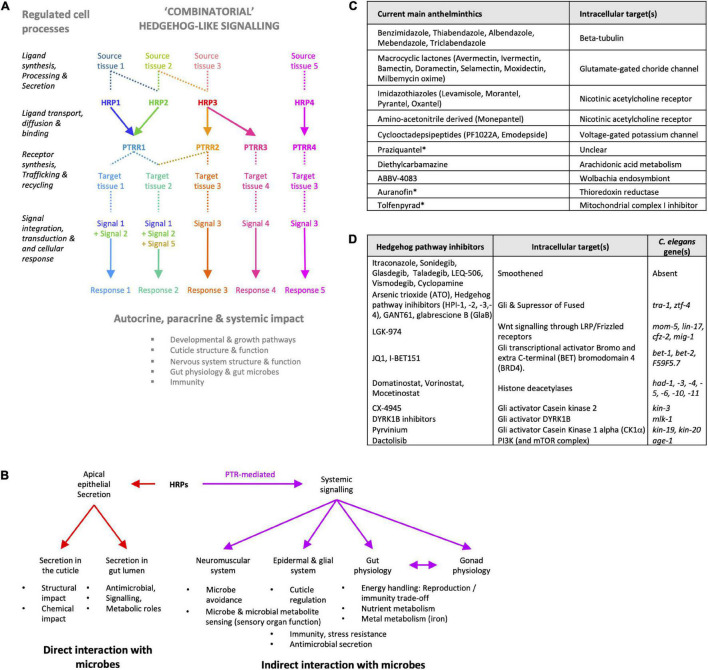
Targeting Hedgehog-like signaling to disrupt nematode-microbe interactions. **(A)** Hedgehog-like signaling (HHLS) involves multiple regulated steps with druggable targets (ligand processing/maturing enzymes, transporters, receptors and co-receptors, regulators of membrane trafficking and recycling) and regulates multiple aspects of nematode physiology (growth, development, immune and structural defenses, nervous system, and gut functions). Based on paralog sequence similarities, overlaps in tissue expression, and transcriptomics data, nematode HHLS may rely on partially redundant and/or combinatorial signaling, to be elucidated. **(B)** Whether and how nematode Hedgehog-related peptides (HRPs) may directly or indirectly interact with microbes also remain to be clearly established. **(C)** The main anthelminthic strategies currently in use or being explored* mostly target the nematode neuromuscular system and do not intersect with HHLS (Elfawal et al., 2019; Hahnel et al., 2020; Partridge et al., 2020). **(D)** Main Hedgehog signaling inhibitors used in oncology either target the less conserved part of the HH pathway (Smoothened and Gli), Wnt signaling, or conserved but low-specificity activators of HH pathway genes (Peer et al., 2019; Jamieson et al., 2020). HH, Hedgehog; HRP, Hedgehog-related peptide; PTRR, Patched-related receptor.

While it has been challenging to demonstrate the existence of a systemic HHLS pathway in *C. elegans* due to the lack of conserved intracellular transducers in nematodes, constitutive intestinal expression of PTRs, such as *ptr-4* and *ptr-24* (Lightfoot et al., 2016; Supplementary Figure 3), suggests that their HRP ligands may be acting systemically. Recent evidence has further helped piece key elements together, now suggesting a major role for HHLS in the development and function of the nematode gut-brain axis (Lin and Wang, 2017). Bacterial methionine deprivation was found to trigger the hypodermal production of the *C. elegans* HRP GRL-21, which systemically inhibited the gut PTR receptor PTR-24, leading to a shift in host lipid metabolism and mitochondrial homeostasis (Lin and Wang, 2017). Our results are consistent with this as we found that exposures to the commensal bacteria *B. subtilis* and *Comamonas* sp. both lead to an up-regulation of *grl-21* (Figure 3C) and transcriptional modulation of lipid metabolism genes (Figure 2B, Supplementary Figure 2, and Supplementary Table 5), while *grl-21* inhibition by RNAi was detrimental to worms exposed to *B. subtilis* and *Comamonas* sp. (they became hypersensitive to thermal stress, Figure 3C). Lipid metabolism is key to both immunity and the HHLS pathway since phospholipid membrane dynamics and cholesterol metabolism are critical to Hedgehog signaling (Cadena Del Castillo et al., 2021). Our data also suggest that *grl-21* contributes to worm resistance to *E. faecalis* infection, together with related HRPs *grl-6, grl-12, grl-22*, and *grl-27* (Figures 3B,C), although they do not point out to specific cognate PTR receptors. Follow-up work on such identified links, notably with genetic epistasis and cell biology approaches, would enable elucidation of *bona fide* pathways, which could initiate the mapping of an elusive HRP ligand-PTR receptor code (Figure 5A).

Such code may also be inferred from cross analysis of gene expression, proteomics and phenomics datasets, where principal component analysis approaches could identify clusters of similarly behaving genes, pointing out to redundancies or receptor/ligand pairings. Current challenges with these approaches remain variability in dataset reporting and access to complete raw datasets, but these limitations are being gradually addressed.

Beyond the proposed emerging role in systemic signaling, the worm HHLS pathway could be mediating host-microbe interactions and gut-brain axis communications by directly or indirectly modulating sensory organ functions, either in glial cells or neurons (Figure 5B). This would impact on the ability of worms to sense microbes and secrete signaling molecules. For instance, down-regulation of the PTR receptor *daf-6* in glial cells was found to enable the reversible remodeling of sensory organs in response to an adverse environment, with impact on serotonin biosynthesis and serotoninergic signaling (Moussaif and Sze, 2009). HHLS could also impact the gut-brain axis via neurogenic pathways, particularly during development. Indeed, inhibition of HHLS genes can lead to: (1) misshapen or dysfunctional chemosensory organ pockets (*che-14, lit-1, daf-6, mom-4/5*) (Michaux et al., 2000; Perens and Shaham, 2005; Liegeois et al., 2007; Oikonomou et al., 2011; Oikonomou and Shaham, 2012), (2) defects in axonal pathfinding (*wrt-8* and *grl-16)* (Riveiro et al., 2017), and (3) defects in neuronal progenitor cell cycle schedule and cell fate (*grl-5*, *grl-7*, *ptr-18*) (Kume et al., 2019; Chiyoda et al., 2021). Thus, as for RNAi effectiveness we initiated genetic knockdown at the late L1 larval stage, some of the effects observed in our study could have resulted from impairment of the late development of the nervous system.

**FIGURE 6:**
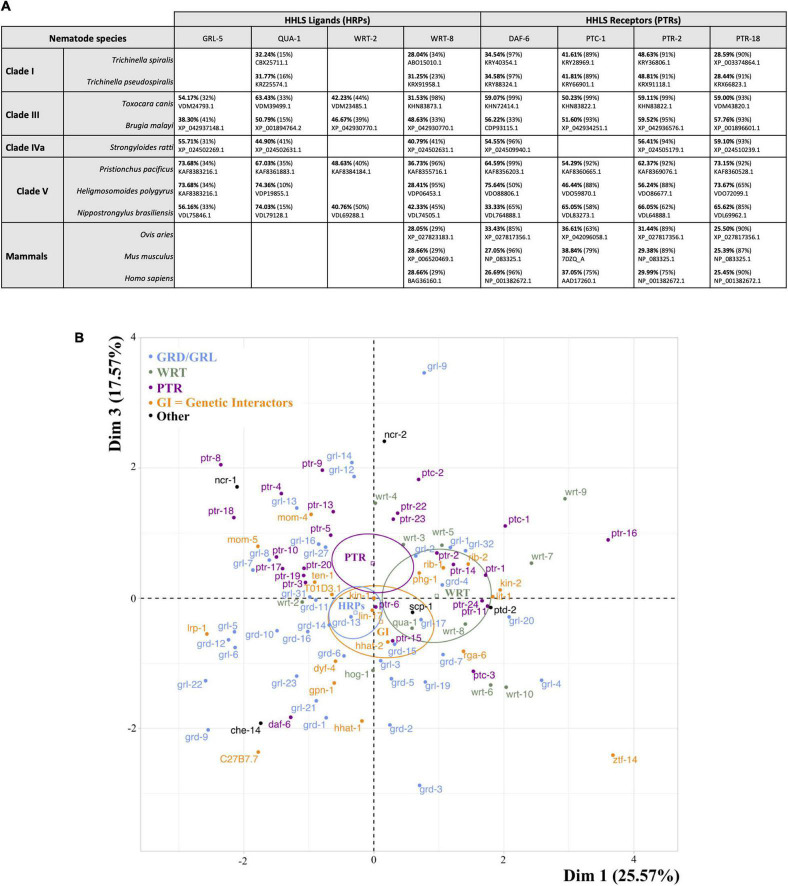
Divergence in sequence identity and differential effects of HHLS pathway gene inhibition on *C. elegans* phenotypes. **(A)** Percentage identity (bold) and percentage of sequence coverage (parentheses) for the most-likely orthologs of selected *C. elegans* HRPs and PTRs across nematode clades and mammals. Accession numbers for proteins are provided below percentage identity. Results were obtained using BLASTP or TBLASTN of *C. elegans* protein against target species databases. Low sequence identities between species suggest that targeting nematode HHLS pathway genes could be a valid strategy for species-specific interventions and may be unlikely to disrupt mammalian host Hedgehog signaling. **(B)** Scaled Principal Component Analysis (PCA) of individuals applied to survival data from *C. elegans* exposed to RNAi targeting HHLS genes (median times of death in stress and infection assays from Figures 3B,C). The plot highlights clusters of RNAi conditions that lead to similar outcomes in the phenotypic assays performed. Dimensions 1 and 3 spread variables in a meaningful manner (Dim 1 against Dim 2 is provided in Supplementary Figure 6). Dim 1 recapitulates variation in stress resistance and separates GRL/GRD from WRT ligands, while Dim 3 spreads data mainly according to resistance to infection and seems to separate PTR genes from reported genetic interactors of the *C. elegans* HHLS pathway (GI). “Other” regroups highly conserved non-PTR genes coding for sterol sensing domain proteins: ptd-2, che-14 (Dispatched orthologs), ncr-1, ncr-2 (NPC1 orthologs), and scp-1 (SCAP ortholog). RNAi clusters may reveal HHLS genes that are co-engaged in a response or a degree of functional redundancy. Overall, effects of individual HRPs and PTRs vary significantly.

Alternatively, HRPs could be directly interacting with microbes, or mediating HHLS directed toward microbes, either directly or indirectly. As secreted peptides, HRPs could act as antimicrobials themselves or direct the production of antimicrobial peptides by target tissues expressing cognate PTRs (Wang et al., 2011). As components of the cuticle (*wrt-2, wrt-8, qua-1*) (Hao et al., 2006c; Liegeois et al., 2006), HRPs may contribute to the cuticle physical and biochemical defenses against microbes. They could also modulate the ability of the epidermis and the cuticle to exert their function of defense against microbes via their role in cuticle and epithelial morphogenesis (Michaux et al., 2000; Zugasti et al., 2005; Hao et al., 2006a,c; Liegeois et al., 2006, 2007). Our enrichment analyses of transcriptomic datasets after microbial exposure also detected hits tagged as “constituent of cuticle” and “cuticle collagen” upon exposure with the “skin” pathogen *M. humicola* (Figure 1). Worms exposed to fungal pathogens attacking the worm intestine: *Harposporium* sp., and epidermis: *D. coniospora* also up-regulated *grl-* and *grd-* HRPs but predominantly down-regulated *wrt-* type HRPs (Figure 3A), possibly highlighting a key distinction between the targets or mode of action of *wrt-* type vs. *grd-/grl-* type HRPs, as our data collectively suggests (Figure 6B). Cuticular HRPs (such as *wrt-2, wrt-8*, and *qua-1* in *C. elegans*) may represent interesting targets to modulate host-microbe interactions for parasitic nematode control, because their disruption may promote microbial adhesion/invasion physically or chemically and/or it may inhibit molting and trigger developmental arrest and death.

Finally, as *C. elegans* investment in reproduction often competes with mounting effective immune responses (TeKippe and Aballay, 2010; Yunger et al., 2017; Anderson and Pukkila-Worley, 2020), HHLS could indirectly impact host-microbe interactions through their role in nematode reproduction, beyond the known developmental role of TRA-1 in sex determination (Hodgkin, 1987; Berkseth et al., 2013; Ji et al., 2021), as shown for *wrt-10*, *ptc-1*, *ptr-2* (Kuwabara et al., 2000; MacNeil et al., 2013; Templeman et al., 2020).

Importantly, the implication of HHLS in nematode-microbe interactions, both commensal and pathogenic, suggests that existing Hedgehog-pathway targeting drugs (Galperin et al., 2019) may be repurposed or evolved to target parasitic roundworms (Figure 5D; Peer et al., 2019; Jamieson et al., 2020). These would complement current anthelmintic strategies, most of which target the nematode neuromuscular system in a way that does not intersect with HHLS (Figure 5C; Elfawal et al., 2019; Hahnel et al., 2020; Partridge et al., 2020). HHLS pathway modulators may synergize with current anthelmintics, improving treatment specificity and efficacy. Moreover, because nematodes exhibit a greater diversity of HRPs and PTRs that also diverges from their hosts (Figure 6A and Supplementary Figure 1; Burglin, 1996, 2008; Burglin and Kuwabara, 2006), it opens the possibility of designing nematode-specific drugs targeting nematode’s HHLS without interfering with the host’s endogenous Hedgehog pathway (Figure 6A). Developing or repurposing such drugs may involve similar phenotypic screening approach as employed in this study, applied to parasitic nematode larvae (Benedetto et al., 2019).

As an alternative to traditional pesticides and anthelminthics, targeting parasitic nematodes by interfering with their ability to adequately manage host-microbe interactions offers new avenues for anthelminthic development that are likely to yield more specific and safer treatments for nematode hosts and for the environment. Before the advent of “omics” and systems biology, such approaches would have relied primarily on serendipitous findings, but we can now explore a fast-expanding database of high-quality datasets with a widening variety of complementary powerful open-source tools to guide the development of future anthelminthics. It is worth noting that this type of “ecological” approaches is well-aligned with the fast-growing trend, within the biopharma industry, to seek new interventions into human diseases (cancers, metabolic, neurodegenerative, and mental health diseases) that primarily target the gut microbiota (Quigley and Gajula, 2020), which presents further opportunities for knowledge and technology transfers between traditionally segregated fields.

## Data Availability Statement

The original contributions presented in the study are included in the article/Supplementary Material, further inquiries can be directed to the corresponding author/s.

## Author Contributions

AB and AZ-P designed the study, prepared the figures, and wrote the manuscript. IA, AB, and AZ-P performed the experiments. IA, AB, AZ-P, MR, and HB analyzed the data. All authors contributed to the article and approved the submitted version.

## Conflict of Interest

The authors declare that the research was conducted in the absence of any commercial or financial relationships that could be construed as a potential conflict of interest.

## Publisher’s Note

All claims expressed in this article are solely those of the authors and do not necessarily represent those of their affiliated organizations, or those of the publisher, the editors and the reviewers. Any product that may be evaluated in this article, or claim that may be made by its manufacturer, is not guaranteed or endorsed by the publisher.

## Footnotes

1
https://www.researchrabbit.ai/

2
https://www.rstudio.com/

3
https://digitalinsights.qiagen.com/
