# Supplementary material for: Meta-Analysis of Caenorhabditis elegans Transcriptomics Implicates Hedgehog-Like Signaling in Host-Microbe Interactions
Source: Front Microbiol. 2022 May 10;13:853629. doi: 10.3389/fmicb.2022.853629 (PMC9127769; doi:10.3389/fmicb.2022.853629)
Supplement: Supplementary file 1 [file Presentation_1.PPTX]

## Slide 1
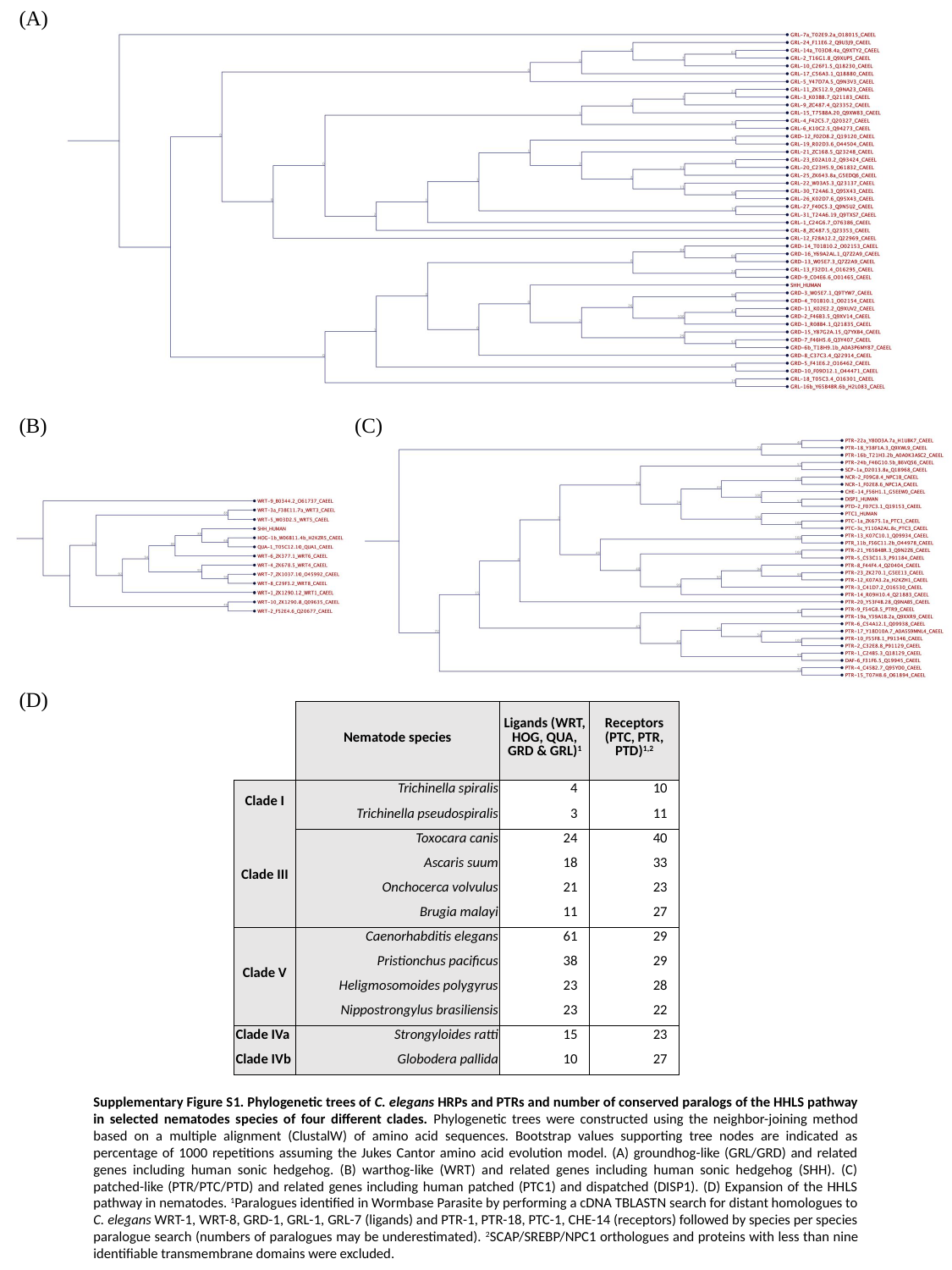

(A)
(B)
(C)
(D)
| | Nematode species | Ligands (WRT, HOG, QUA, GRD & GRL)1 | Receptors (PTC, PTR, PTD)1,2 |
| --- | --- | --- | --- |
| Clade I | Trichinella spiralis | 4 | 10 |
| | Trichinella pseudospiralis | 3 | 11 |
| Clade III | Toxocara canis | 24 | 40 |
| | Ascaris suum | 18 | 33 |
| | Onchocerca volvulus | 21 | 23 |
| | Brugia malayi | 11 | 27 |
| Clade V | Caenorhabditis elegans | 61 | 29 |
| | Pristionchus pacificus | 38 | 29 |
| | Heligmosomoides polygyrus | 23 | 28 |
| | Nippostrongylus brasiliensis | 23 | 22 |
| Clade IVa | Strongyloides ratti | 15 | 23 |
| Clade IVb | Globodera pallida | 10 | 27 |
Supplementary Figure S1. Phylogenetic trees of C. elegans HRPs and PTRs and number of conserved paralogs of the HHLS pathway in selected nematodes species of four different clades. Phylogenetic trees were constructed using the neighbor-joining method based on a multiple alignment (ClustalW) of amino acid sequences. Bootstrap values supporting tree nodes are indicated as percentage of 1000 repetitions assuming the Jukes Cantor amino acid evolution model. (A) groundhog-like (GRL/GRD) and related genes including human sonic hedgehog. (B) warthog-like (WRT) and related genes including human sonic hedgehog (SHH). (C) patched-like (PTR/PTC/PTD) and related genes including human patched (PTC1) and dispatched (DISP1). (D) Expansion of the HHLS pathway in nematodes. 1Paralogues identified in Wormbase Parasite by performing a cDNA TBLASTN search for distant homologues to C. elegans WRT-1, WRT-8, GRD-1, GRL-1, GRL-7 (ligands) and PTR-1, PTR-18, PTC-1, CHE-14 (receptors) followed by species per species paralogue search (numbers of paralogues may be underestimated). 2SCAP/SREBP/NPC1 orthologues and proteins with less than nine identifiable transmembrane domains were excluded.
