# Supplementary material for: Meta-Analysis of Caenorhabditis elegans Transcriptomics Implicates Hedgehog-Like Signaling in Host-Microbe Interactions
Source: Front Microbiol. 2022 May 10;13:853629. doi: 10.3389/fmicb.2022.853629 (PMC9127769; doi:10.3389/fmicb.2022.853629)
Supplement: Supplementary file 2 [file Presentation_2.PPTX]

## Slide 1
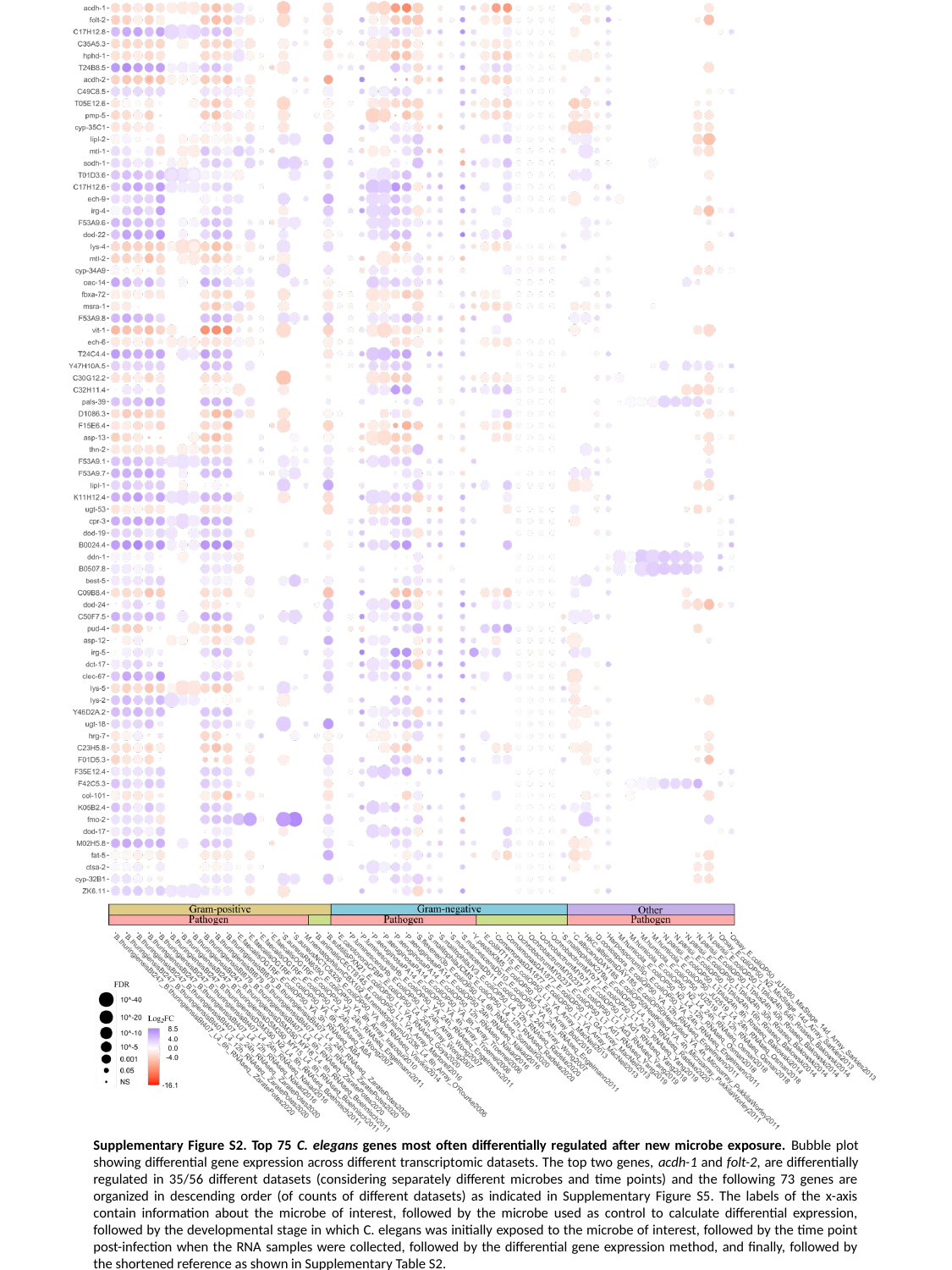

Supplementary Figure S2. Top 75 C. elegans genes most often differentially regulated after new microbe exposure. Bubble plot showing differential gene expression across different transcriptomic datasets. The top two genes, acdh-1 and folt-2, are differentially regulated in 35/56 different datasets (considering separately different microbes and time points) and the following 73 genes are organized in descending order (of counts of different datasets) as indicated in Supplementary Figure S5. The labels of the x-axis contain information about the microbe of interest, followed by the microbe used as control to calculate differential expression, followed by the developmental stage in which C. elegans was initially exposed to the microbe of interest, followed by the time point post-infection when the RNA samples were collected, followed by the differential gene expression method, and finally, followed by the shortened reference as shown in Supplementary Table S2.
