# Supplementary material for: Meta-Analysis of Caenorhabditis elegans Transcriptomics Implicates Hedgehog-Like Signaling in Host-Microbe Interactions
Source: Front Microbiol. 2022 May 10;13:853629. doi: 10.3389/fmicb.2022.853629 (PMC9127769; doi:10.3389/fmicb.2022.853629)
Supplement: Supplementary file 3 [file Presentation_3.PPTX]

## Slide 1
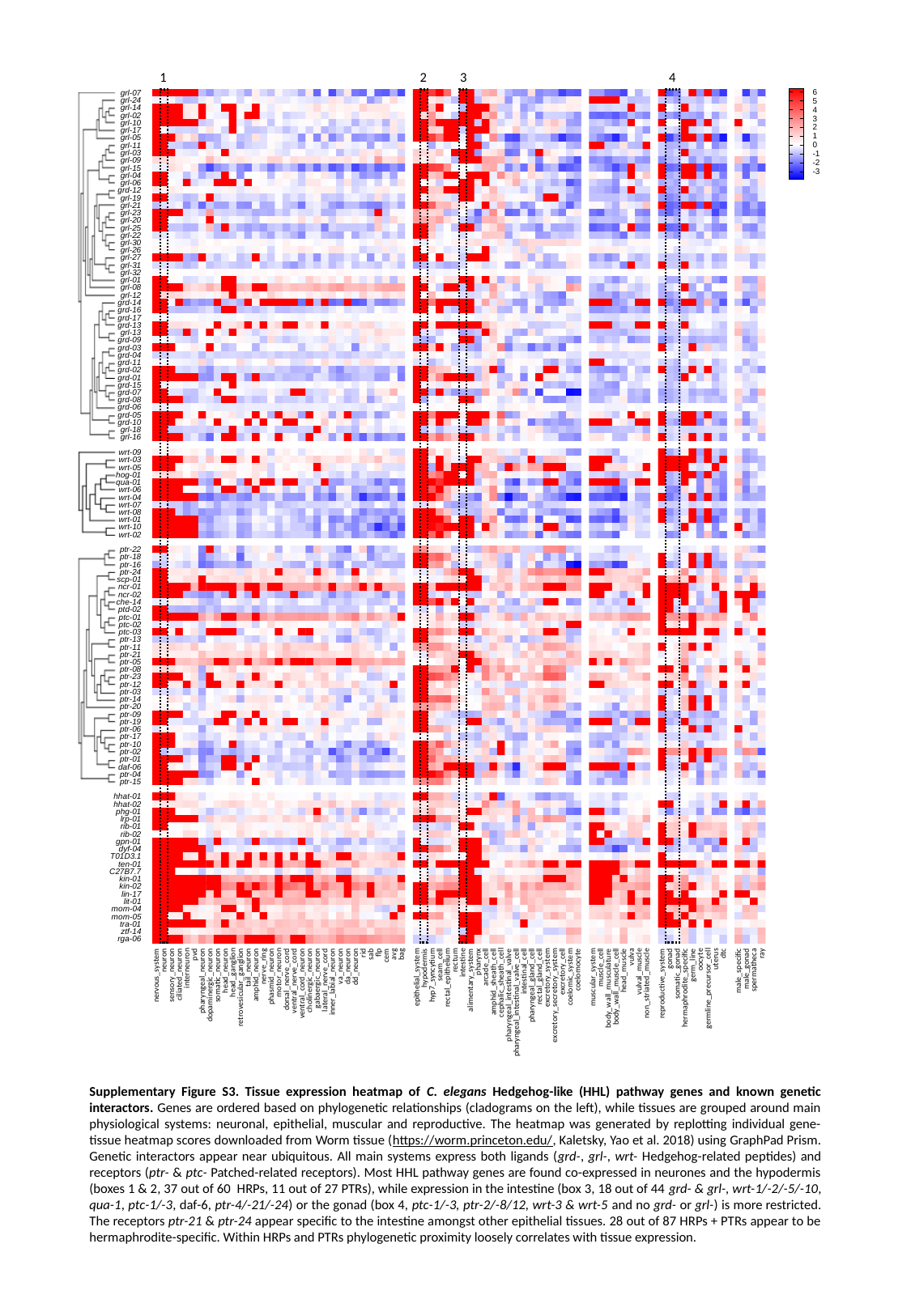

1
2
3
4
Supplementary Figure S3. Tissue expression heatmap of C. elegans Hedgehog-like (HHL) pathway genes and known genetic interactors. Genes are ordered based on phylogenetic relationships (cladograms on the left), while tissues are grouped around main physiological systems: neuronal, epithelial, muscular and reproductive. The heatmap was generated by replotting individual gene-tissue heatmap scores downloaded from Worm tissue (https://worm.princeton.edu/, Kaletsky, Yao et al. 2018) using GraphPad Prism. Genetic interactors appear near ubiquitous. All main systems express both ligands (grd-, grl-, wrt- Hedgehog-related peptides) and receptors (ptr- & ptc- Patched-related receptors). Most HHL pathway genes are found co-expressed in neurones and the hypodermis (boxes 1 & 2, 37 out of 60 HRPs, 11 out of 27 PTRs), while expression in the intestine (box 3, 18 out of 44 grd- & grl-, wrt-1/-2/-5/-10, qua-1, ptc-1/-3, daf-6, ptr-4/-21/-24) or the gonad (box 4, ptc-1/-3, ptr-2/-8/12, wrt-3 & wrt-5 and no grd- or grl-) is more restricted. The receptors ptr-21 & ptr-24 appear specific to the intestine amongst other epithelial tissues. 28 out of 87 HRPs + PTRs appear to be hermaphrodite-specific. Within HRPs and PTRs phylogenetic proximity loosely correlates with tissue expression.
