# Supplementary material for: Meta-Analysis of Caenorhabditis elegans Transcriptomics Implicates Hedgehog-Like Signaling in Host-Microbe Interactions
Source: Front Microbiol. 2022 May 10;13:853629. doi: 10.3389/fmicb.2022.853629 (PMC9127769; doi:10.3389/fmicb.2022.853629)
Supplement: Supplementary file 4 [file Presentation_4.PPTX]

## Slide 1
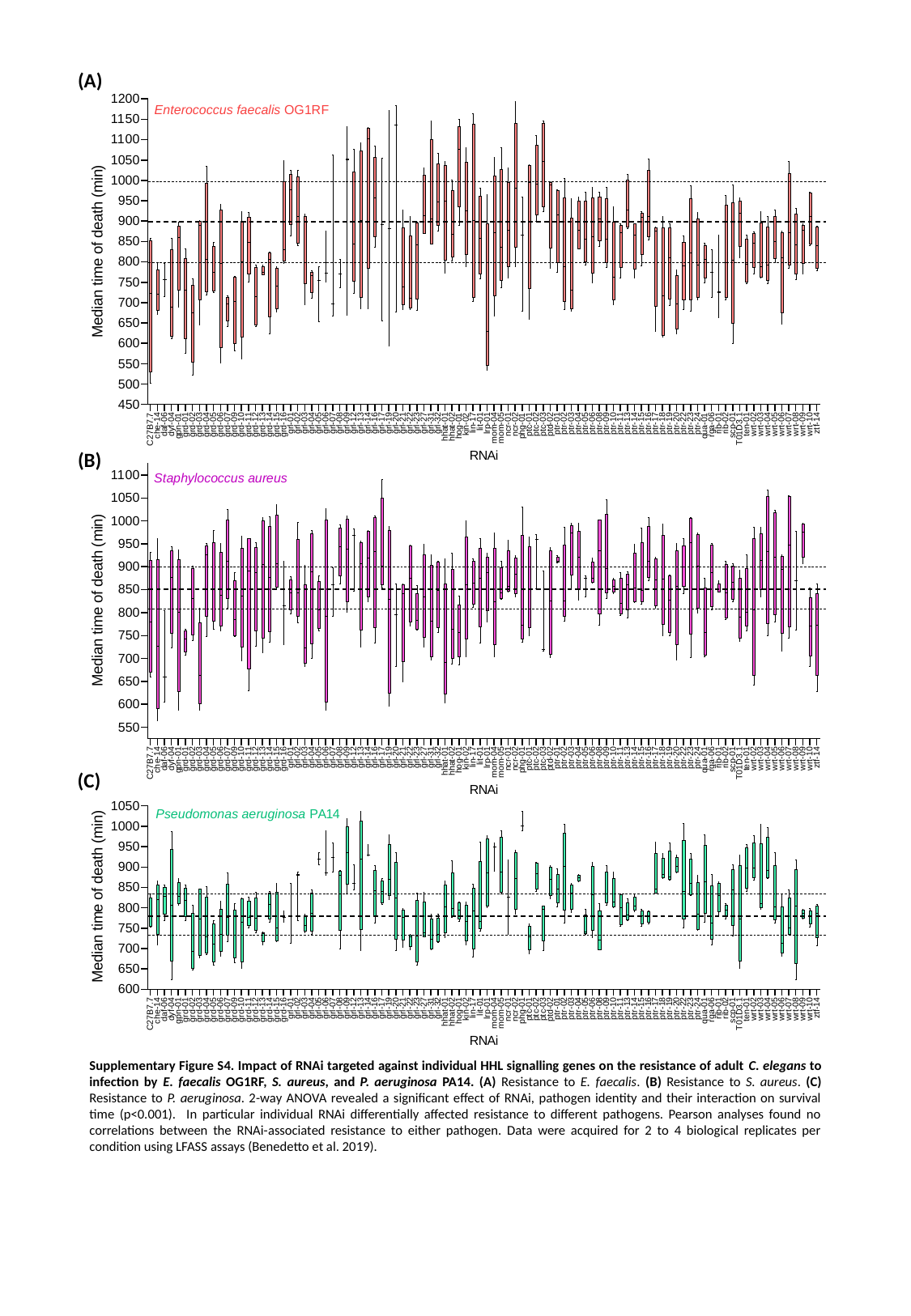

(A)
(B)
Supplementary Figure S4. Impact of RNAi targeted against individual HHL signalling genes on the resistance of adult C. elegans to infection by E. faecalis OG1RF, S. aureus, and P. aeruginosa PA14. (A) Resistance to E. faecalis. (B) Resistance to S. aureus. (C) Resistance to P. aeruginosa. 2-way ANOVA revealed a significant effect of RNAi, pathogen identity and their interaction on survival time (p<0.001). In particular individual RNAi differentially affected resistance to different pathogens. Pearson analyses found no correlations between the RNAi-associated resistance to either pathogen. Data were acquired for 2 to 4 biological replicates per condition using LFASS assays (Benedetto et al. 2019).
(C)
