# Supplementary material for: Meta-Analysis of Caenorhabditis elegans Transcriptomics Implicates Hedgehog-Like Signaling in Host-Microbe Interactions
Source: Front Microbiol. 2022 May 10;13:853629. doi: 10.3389/fmicb.2022.853629 (PMC9127769; doi:10.3389/fmicb.2022.853629)
Supplement: Supplementary file 5 [file Presentation_5.PPTX]

## Slide 1
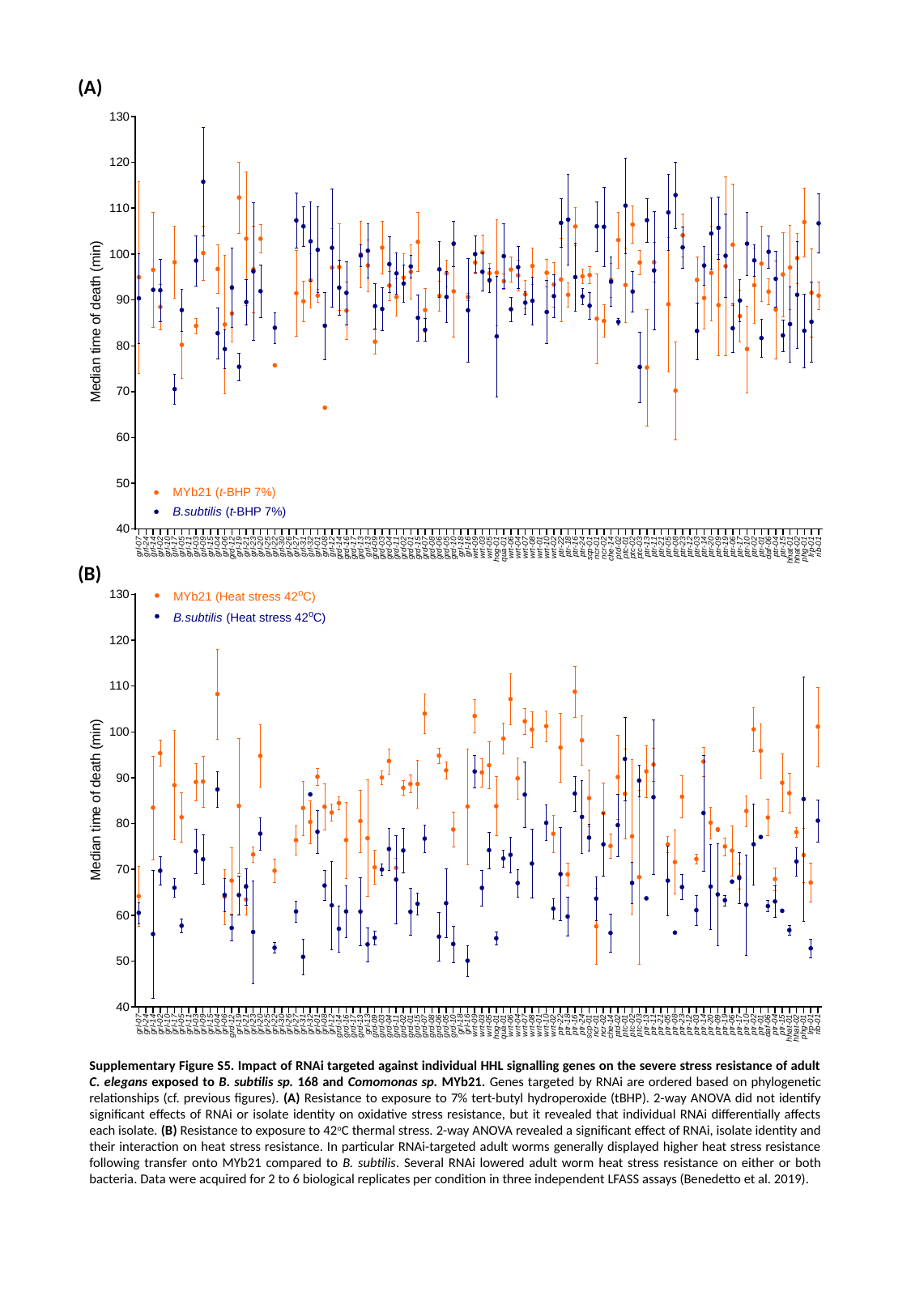

(A)
(B)
Supplementary Figure S5. Impact of RNAi targeted against individual HHL signalling genes on the severe stress resistance of adult C. elegans exposed to B. subtilis sp. 168 and Comomonas sp. MYb21. Genes targeted by RNAi are ordered based on phylogenetic relationships (cf. previous figures). (A) Resistance to exposure to 7% tert-butyl hydroperoxide (tBHP). 2-way ANOVA did not identify significant effects of RNAi or isolate identity on oxidative stress resistance, but it revealed that individual RNAi differentially affects each isolate. (B) Resistance to exposure to 42oC thermal stress. 2-way ANOVA revealed a significant effect of RNAi, isolate identity and their interaction on heat stress resistance. In particular RNAi-targeted adult worms generally displayed higher heat stress resistance following transfer onto MYb21 compared to B. subtilis. Several RNAi lowered adult worm heat stress resistance on either or both bacteria. Data were acquired for 2 to 6 biological replicates per condition in three independent LFASS assays (Benedetto et al. 2019).
