# Supplementary material for: Meta-Analysis of Caenorhabditis elegans Transcriptomics Implicates Hedgehog-Like Signaling in Host-Microbe Interactions
Source: Front Microbiol. 2022 May 10;13:853629. doi: 10.3389/fmicb.2022.853629 (PMC9127769; doi:10.3389/fmicb.2022.853629)
Supplement: Supplementary file 6 [file Presentation_6.PPTX]

## Slide 1
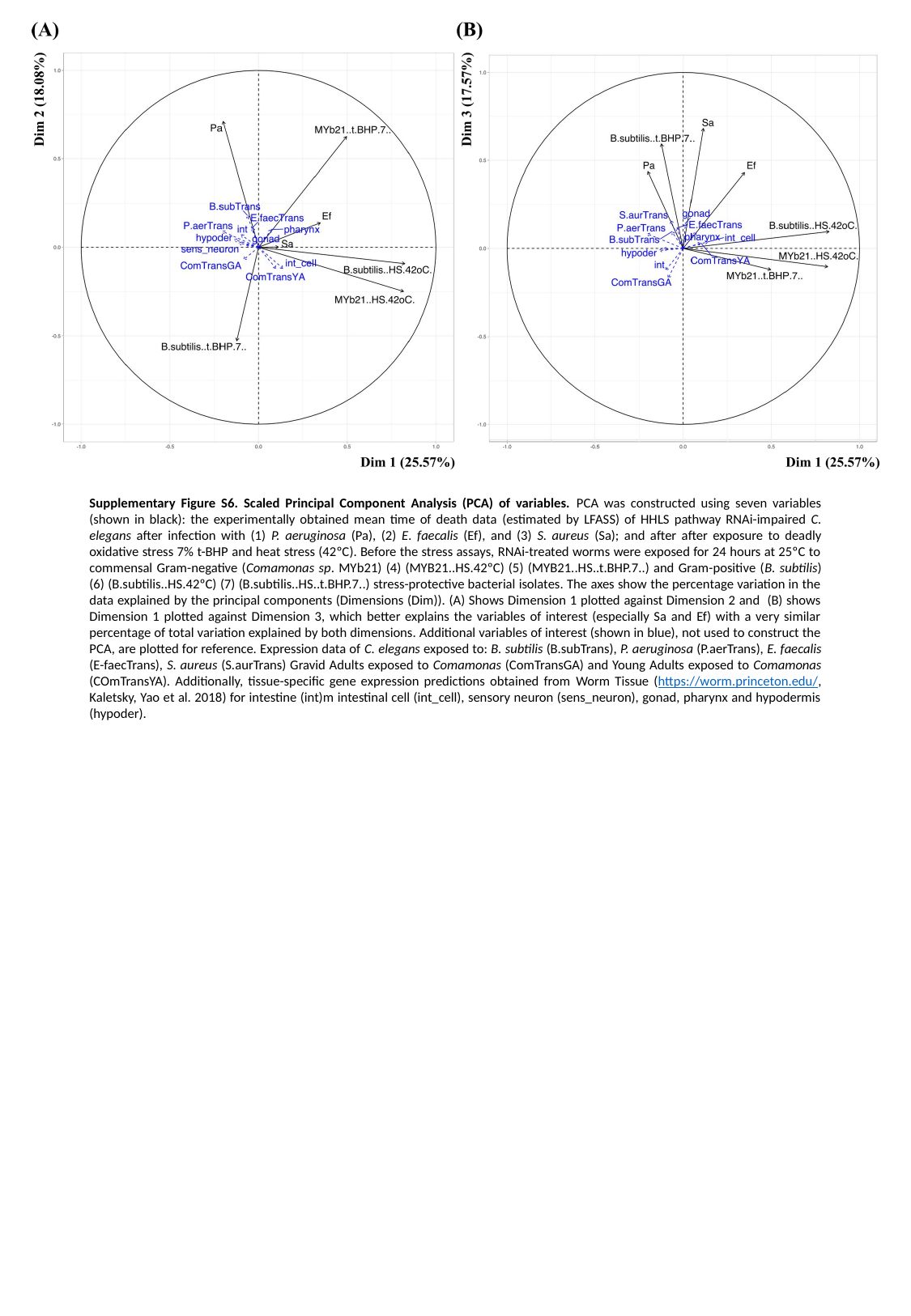

Supplementary Figure S6. Scaled Principal Component Analysis (PCA) of variables. PCA was constructed using seven variables (shown in black): the experimentally obtained mean time of death data (estimated by LFASS) of HHLS pathway RNAi-impaired C. elegans after infection with (1) P. aeruginosa (Pa), (2) E. faecalis (Ef), and (3) S. aureus (Sa); and after after exposure to deadly oxidative stress 7% t-BHP and heat stress (42ºC). Before the stress assays, RNAi-treated worms were exposed for 24 hours at 25ºC to commensal Gram-negative (Comamonas sp. MYb21) (4) (MYB21..HS.42ºC) (5) (MYB21..HS..t.BHP.7..) and Gram-positive (B. subtilis) (6) (B.subtilis..HS.42ºC) (7) (B.subtilis..HS..t.BHP.7..) stress-protective bacterial isolates. The axes show the percentage variation in the data explained by the principal components (Dimensions (Dim)). (A) Shows Dimension 1 plotted against Dimension 2 and (B) shows Dimension 1 plotted against Dimension 3, which better explains the variables of interest (especially Sa and Ef) with a very similar percentage of total variation explained by both dimensions. Additional variables of interest (shown in blue), not used to construct the PCA, are plotted for reference. Expression data of C. elegans exposed to: B. subtilis (B.subTrans), P. aeruginosa (P.aerTrans), E. faecalis (E-faecTrans), S. aureus (S.aurTrans) Gravid Adults exposed to Comamonas (ComTransGA) and Young Adults exposed to Comamonas (COmTransYA). Additionally, tissue-specific gene expression predictions obtained from Worm Tissue (https://worm.princeton.edu/, Kaletsky, Yao et al. 2018) for intestine (int)m intestinal cell (int_cell), sensory neuron (sens_neuron), gonad, pharynx and hypodermis (hypoder).
